# The mediating functions of coping strategies for the relationship between the loss of personal resources during COVID-19 and the providing support to students: The differences between believing and non-believing teachers in Poland

**DOI:** 10.1371/journal.pone.0327434

**Published:** 2025-07-17

**Authors:** Krzysztof Jurek, Iwona Niewiadomska, Joanna Chwaszcz, Monika Dobrogowska

**Affiliations:** 1 Department of Sociology of Culture, Religion and Social Participation, John Paul II Catholic University of Lublin, Lublin, Poland; 2 Department of Social Psychoprevention, John Paul II Catholic University of Lublin, Lublin, Poland; Bangladesh Open University, BANGLADESH

## Abstract

**Introduction:**

The teaching profession is not only related to the transmission of knowledge and the formation of attitudes in alumni, but also to the provision of multidimensional support to students in difficult/problematic situations. Social support refers to the provision of psychological and material resources by the social network to enhance the individual’s capacity to cope with stress.

**Aim:**

The aim of this article is to present the mediating functions of stress coping strategies for the relationships occurring between the severity of stress generated by losses in personal resources during the COVID-19 pandemic and providing support to students in believing and non-believing teachers.

**Methods:**

The nationwide and representative study was conducted from September 13, 2021 to October 1, 2021. The study included a total of 2500 teachers. The mean age was 43.93 years (SD = 9.89). The Polish adaptation of Hobfoll’s Conservation of Resources-Evaluation (COR-E), the Inventory for Measuring Coping with Stress (MINI-COPE) and The Berlin Social Support Scales (BSSS) were employed in the research.

**Results:**

There are statistically significant differences between believing and non-believing teachers in the mediating functions of stress coping strategies for the relationship between the perception of personal losses and the providing support to students. Teachers’ religiosity promotes greater flexibility in the use of coping strategies in the process of coping with chronic stress compared to non-believing teachers.

**Conclusion:**

The results presented justify the postulate that teachers should benefit from various forms of support to counteract personal losses, including support of a religious nature.

## Introduction

### Providing support to students

The analysis of the presented objective stems from the fact that the profession of teaching is not only related to the transmission of knowledge and the formation of attitudes in alumni, but also to the providing support to students in difficult/problematic situations [1–3]. The providing support to students is evidenced by the relationship between the teacher (support person) and the student (supported person) when there is a process of exchange of emotions, information, instruments of action and/or material goods between the mentioned actors. The emotional support provided consists of conveying reassuring emotions, increasing self-esteem and reflecting care, concern and a sense of belonging. The provision of informational support is related to the transmission of messages for a better understanding of the life situation and/or the problem at hand, as well as the provision of feedback on the effectiveness of the supported person’s remedial actions. The instrumental support provided is a kind of instruction by informing about specific ways to deal with a particular situation, which is a form of modelling effective coping strategies in dealing with problems and/or achieving desired goals. In-kind (material) support, on the other hand, refers to the provision of material, in-kind and/or financial assistance to a person in need [4–5]. The informational, emotional, instrumental and/or material support provided often has a buffering effect on the assisted persons in that it helps to reduce the severity of their stress and/or protect them from the occurrence of negative consequences of psychological tension on their physiological, mental and/or social functioning [6–11].

The mechanisms characteristic of social support provided are confirmed by the phenomenon of stress crossover, which involves the flow and mutual exchange of resources in the process of coping with stress. The analysis of the providing support to students, on the one hand, to the great importance of the interpersonal context in overcoming the difficulties encountered through the mechanism of giving/providing assistance by persons characterised by a higher level of resources determining greater resilience, and, on the other hand, to the formation of a mechanism of demand for a specific form of support in weaker persons [12]. The mechanisms presented also support the finding that the development of individual resilience to stress is highly dependent on the interaction of various social systems, which, through supportive relationships (e.g., providing a sense of security), shape the course of individual development and the learning of adaptive behaviour in various stressful situations [13].

The teachers, as significant others, often provide support to students in coping with stress and thus protect the mental health of their pupils, e.g., through emotionally supportive behaviour, empathy, clarifying events (e.g., as a result of acting as mentors), modelling constructive coping strategies (e.g., through sport, music), creating challenges, generating situations for success and/or creating circumstances for managing failures. And the effectiveness of the measures taken is high when the teacher support offered, on the one hand, meets the needs of students experiencing stress and, on the other hand, creates a coherent network of connections with other support systems [14–16].

Teachers providing support to students may experience negative consequences of initiating and/or maintaining supportive relationships. One of these may be the phenomenon of professional burnout involving experiencing negative symptoms of emotional, mental and/or physical exhaustion [14]. The literature suggests that there is a negative relationship between experiencing teacher burnout and the support provided to students [17]. In the context of the relationship presented, it should also be emphasised that professional burnout syndrome is a common phenomenon among teachers. Its prevalence (depending on the study) was found to vary between 11 and 50% of the teacher population [18–19].

The previous research supports allows the conclusion to be drawn that the risk of job exhaustion increases when there are few resilience resources, i.e., when teachers have few resources (e.g., low self-efficacy, lack of professional appreciation, inadequate working conditions, faulty communication in the working environment, staff conflicts, low pay) and/or when they lose the resources they have (e.g., professional autonomy, social support they receive, materials needed for their work) [20–24].

### Conservation of resources theory and coping strategies

Conservation of resources theory (COR) also indicates that experiencing resource losses – e.g., in the form of perceived threats of losses in key/central resources and/or the occurrence of real/actual losses in key/central resources and/or the inability to acquire key/central resources while expending a great deal of effort – leads to the occurrence of stress [25]. The regularities presented justify the conclusion that the occurrence of resource losses is characteristic of the experience of different types of stress-generating difficulties – e.g., deprivation of needs, feelings of threat, physical and/or mental suffering, overload, internal and/or external conflicts, inconvenience, new situations. These difficulties may co-occur and/or overlap both at a specific moment in time and generate chronic stress, which involves the long-term experience of emotional tension of a negative nature. Unlike other crises or disasters, the COVID-19 pandemic led to a long-term situation in which entire societies/individuals making up the general population have to face a series of stressful events due to the existence of an epidemiological threat over a long period of time – because of uncertainty, change in lifestyle, social isolation, psychological and/or physical burden, financial pressure, among others [10]. The findings also confirm the occurrence of high resource losses during the COVID-19 pandemic. Significant risk factors for psychological difficulties (mainly symptoms: depression, anxiety and/or post-traumatic stress disorder) during the pandemic included unfavourable distribution of key resources (low levels of resources and/or experiencing resource losses) – including personal infection, being in quarantine, feeling stigmatised due to the onset of infection, loss of work, loss of income, previous psychological problems, low economic status, high intensity of interpersonal conflicts, lack of formal relationships with other people, low levels of social relationships, perception of low quality of one’s life, lack of a sense of rootedness, lack of social support [26–31].

The mechanisms described in COR theory also justify the position that it is not individual resources, but whole packs/caravans of resources that perform regulatory functions in human functioning. The pooling of resources into caravans (their co-occurrence) is due to the fact that they are the result of repeated experiences in different spheres of functioning throughout life in the form of mental representations [32–35]. A specific caravan is constituted by personal resources, which are constituted, among others, by resource categories such as a sense of control over one’s own life, organisational skills, communication skills, optimism, self-efficacy and religiosity. The caravan/bundle of personal resources performs a variety of functions – mainly in terms of resilience to experienced stress, managing one’s own life and/or directing events in the surrounding reality. The regulatory functions of the personal resources are due to the fact that their presence influences one’s involvement in the actions undertaken, sustains motivation in their implementation, generates belief in the possibility of achieving the desired goal, fosters the occurrence of control over one’s own behaviour and the surrounding reality, reduces fear of the future and/or strengthens positive self-esteem. According to the first principle of COR theory – referred to as the primacy of loss principle – resource losses (including personal losses) are strongly felt by those experiencing them.

The perception of resource depletion fosters the fact that people begin to prefer more defensive ways of coping, less likely to use problem-solving-oriented strategies in favour of a preference for evasive and/or emotional reactions. The most adverse circumstances occur when the phenomenon of resource depletion occurs. According to the fourth COR principle – referred to as the desperation principle – a very low level of resources generates defensive behaviour in order for a person to preserve/protect his or her self. Consequently, his or her behaviour often becomes defensive, passive, aggressive and/or irrational [33]. The presented regularities are exemplified by the results of research relating to the relationship between resource distribution and occupational functioning. Namely, it was found that a high level of personal resources significantly co-occurs with high professional functioning effectiveness. Whereas a decrease in personal resources contributes to a decrease in professional functioning effectiveness [23,33].

Factors that may modify the relationship between perceived resource losses and human behaviour include stress coping strategies. The activities in problematic situations may serve the purpose of: a) solving the difficulties encountered, i.e., changing the situation through the use of active/task-oriented coping strategies, b) maintaining emotional balance so that psychological resilience and/or social functioning does not break down – passive (emotional and/or avoidant) coping strategies are used for this purpose. The correlations presented indicate that coping with stress is effective when a person constructively resolves the difficulties that arise, but at the same time maintains mental balance. It should also be emphasised that so far it has not been clearly established which coping strategies are most effective in dealing with stress. Some analyses tend to conclude that the effectiveness of coping strategies depends on the situational context. In contrast, others show that even when the situational context is taken into account, active strategies have greater adaptive value relative to passive actions – both evasive and emotional [34].

### Religiosity as a personal resource

An important issue is the search for resources that protect individuals who are particularly vulnerable to the occurrence of stress during the COVID 19 pandemic. The results of the study convince us that an important protective factor against the negative effects of stress is religiosity, which in this article is understood as a personal resource resulting from an established tradition for religious beliefs, practices (private and/or public with a group of co-religionists) and rituals related to Transcendence/Higher Power [35–37].

Faith can be seen as a personal resource that protects against the negative effects of stress for two important reasons. Firstly, religious faith is closely linked to a person’s sense of meaning in life. Thus, religiosity situated in a system of intrinsic values contributes to the fact that the choices made by the human individual are directed towards such goals that, in his personal perception, constitute an essential value and thus give meaning to his existence. In this view, religiosity can be an important factor that mobilises a person to engage in intentional behaviour. And its high intensity may lead a person to be more likely to seek constructive solutions even in the most unfavourable circumstances, to treat experienced setbacks as challenges rather than failures and/or to have a greater willingness to take pro-social action. Secondly, religiosity constituting internal standards represents a kind of internal pressure to embody the values and norms of behaviour that result from these standards. Its regulatory function consists, among other things, in the fact that adherence to religious values and norms leads to satisfaction and increased self-respect, while breaking them leads to remorse and lowered self-esteem. The self-regulatory mechanism outlined is responsible for the direction and consistency of actions taken, on the one hand, and protects against the negative consequences of stress, on the other hand [38–43]. The results indicate that negative religious coping is more strongly linked to COVID-19 anxiety than positive religious coping. The literature suggests that coping strategies play a significant role in shaping how well an individual adjusts to stressful circumstances. Positive religious coping acts as a protective factor against negative psychological effects in individuals with a strong religious affiliation or identity and close connections to their religious communities. The personal resource gains associated with choosing turning to religion in stressful situations stem from seeking security and hope in faith, as well as engaging in religious practices such as prayer and/or meditation [44–45]. Meta-analyses indicated the relationship between positive religious coping and flourishing (well-being) [46]. Religiousness was a commonly reported coping mechanism with varying levels of favorable associations with mental health and COVID-19-related behaviors [47]. On the other hand, research conducted in Poland (using the MINI-COPE) shows that a turn towards religion correlates negatively with teachers‘well-being and self-efficacy (Teachers’ Well-Being Scale; SNN) [48–49]. In other polish studies, turning to religion did not correlate with life satisfaction and resilience in teachers [50] A study of university teachers in Poland, found that the use of active and emotion-focused strategies (within this strategy, the authors included a turning to religion, MINI-COPE) did not affect the relationship between stress and professional burnout and chronic fatigue [51]. Research on coping often suffers from a lack of solid theoretical grounding, consistent methodology, and clear operational definitions—particularly when accounting for cultural, linguistic, and contextual variations in how coping behaviors manifest. This becomes especially apparent in the case of constructs like religion, which are variably categorized as adaptive, maladaptive, or neutral, leading to inconsistency and ambiguity in their interpretation [52].

Based on the literature review presented, the following hypotheses were put forward:

**Hypothesis 1.** Among teachers, an increase in stress generated by personal losses is significantly associated with lower levels of support provided to students.

**Hypothesis 2.** Among teachers, coping strategies are mediators for the relationship between the severity of stress generated by personal losses and the intensity of support provided to students.

**Hypothesis 3.** In the group of non-believing teachers relative to the group of believing teachers, other coping strategies mediate the relationship between the intensity of stress generated by personal losses and the intensity of support provided to students.

## Materials and methods

### Measures

#### Conservation of Resources (COR) evaluation.

The dynamics of resource conservation were measured using the Polish adaptation of Stevan E. Hobfoll’s Conservation of Resources evaluation questionnaire (COR evaluation) [53]. The questionnaire contains a list of 74 resources. In the first step, the respondents rated each resource on a 5-point scale where 1 means not at all and 5 means, to a very large extent, in2 categories: loss and gain (To what extent have I gained these resources in my life?/To what extent have I lost these resources in my life?). In the second step, the respondents assigned values to each resource, where “1” means no value and“5” a very great value (How important are the following resources to me?). Various survey resources were taken into account, e.g., “Family stability,” “Good relations with my children,” “My children’s health,” “A sense of closeness with my spouse or partner,” “Awareness of the goal to which I am going in life,” “Belonging to an organization where I can share my interests with others.” The Cronbach’s alpha coefficient in the study was 0.78 for the loss of personal resources, 0.70 for the profit of personal resources and 0.73 for the importance of personal resources, respectively. In this study, we analyzed only one domain — loss of personal resources. The mean score was 12.16 (SD = 6.93)

#### Mini-COPE.

The Polish version of the Mini-COPE inventory was used to measure coping strategies. It is a shortened version of the Multimodal Inventory for Measurement of Coping with Stress-COPE [54] and measures coping in terms of disposition. It consists of 28 statements that are part of 14 strategies for coping with stress, including active coping, planning, positive revalidation, acceptance, sense of humor, turning to religion, seeking emotional support, seeking instrumental support, taking care of something else, denial, discharge, use of psychoactive substances, cessation of activities, and self-blaming. There are two theorems for each strategy. Mini-COPE can also be divided into 7 factors, i.e., Active Coping which includes the following strategies: Active coping, Planning, Positive reinterpretation; Seeking Support including Seeking emotional support and Seeking instrumental support; Helplessness including: Using psychoactive substances, Restraint coping, Self-blame; and Avoidance Behaviours including Finding other activities, Denial and Discharge, Turning to Religion, Acceptance, and Humour were treated as independent factors. The tested respondent refers to each statement by marking one possible answer on a four-point scale where 0—means “I almost never do so” and 3—means “I almost always do so”. The obtained psychometric properties are satisfactory. The half-reliability in the study for 14 scales was 0.86 (Guttman’s index 0.87). The mean scores and standard deviations in this study were as follows: active coping (M = 2.43, SD = 0.54), helplessness (M = 1.03, SD = 0.51), seeking support (M = 2.11, SD = 0.66), avoiding (M = 1.75, SD = 0.55), acceptance (M = 2.13, SD = 0.55), humour (M = 1.27, SD = 0.69), and turning to religion (M = 1.49, SD = 1.03).

#### Provided Support (BSSS).

Berlin Social Support Scales (BSSS) [55] are self-report measures to assess perceived available support, need for support, support seeking, actually received support and protective buffernig support. The Cronbach’s alpha coefficient in the study was 0.78 for perceived social support, 0.83 for received social support, 0.81 for need for support; 0.72 for support seeking, 0.78 for protective buffering, 0.82. for provided social support. Participants indicate their agreement with the statements on a four-point Likert-type scale. Possible endorsements are strongly disagree – 1, somewhat disagree – 2, somewhat agree – 3 and strongly agree – 4. Negative items need to be reversed. Scale scores are obtained either by adding up item responses (sum scores) or by generating the scale mean score. In this study, we analyzed only one domain — provided support. The domain consists of 14 items, i.e., “I showed him how much I cherish and accept him”, “I helped him find something positive in his situation”, “ I encouraged him not to give up”. The mean value for provided support was 3.21 (SD = 0.50)

### Participants

The nationwide survey was conducted from September 13, 2021 to October 1, 2021. The sampling scheme included two stages. In the first stage, it was assumed to draw a minimum of 1,200 schools from all over Poland (16 main administrative regions, voivodeships). A stratified random sampling design was used in this study to improve the sample’s representativeness. The selection of schools was carried out on the basis of publicly available data collected from the Register of Schools and Educational Institutions (https://rspo.gov.pl/). The research sample embraced 1228 units, including 868 elementary schools and 333 post-primary schools. A random sampling was then performed based on the selection of the appropriate number of schools of a given type for each region in Poland using a pseudo-random number generator (PRNG). This procedure allowed the identification of specific units from which all teachers could participate in the survey (each person included in a given group had a non-zero probability of taking part in the survey). After the first stage of the survey, 15286 correctly completed questionnaires were obtained. Only teachers who were willing to give verbal consent to participate in the study were recruited. To ensure the representativeness of the results, the next sampling draw was conducted. The draw sampling included control of the following characteristics: place of residence (namely the region of Poland), seniority and professional situation of teachers. Finally, the sample included 2500 observations.

The study included 2042 women and 458 men, aged between 24 and 76 years. The mean age was 43.93 years (SD = 9.89). The average seniority was 18.85 years (SD = 11.22). There were 71.35 believers and 5.1% non-believers in the study group. About a quarter of the respondents refused to answer a question about faith. Table 1 presents the socio-demographic characteristics of the respondents in the study.

**Table 1:** Participant Demographics.

Variable	Percentage or Mean [N=2500]	
Gender	Men	18.3
	Women	81.7
Age		43.9
Seniority		18.9
Degree teacher	Trainee teacher	6.2
	*Contract teacher*	19.0
	Appointed teacher	19.4
	*Certified teacher*	55.5
Place of residence	Urban area	64.0
	Rural area	36.0
Declaration of faith	Believers	71.3
	Non-believers	5.1
	Refusal to answer	23.6
To what extent does the current situation related to the COVID-19 pandemic limit your social and community activities?	Not at all	18.0
	Slightly	25.7
	Moderately	33.9
	Extremely	20.5
	Makes activity impossible	2.0
To what extent does the current situation related to the COVID-19 pandemic limit your social and work activities?	Not at all	26.6
	Slightly	27.8
	Moderately	29.5
	Extremely	15.1
	Makes activity impossible	1.1
Degree of professional advancement	Trainee Teacher	6.2
	Contracted Teacher	19.0
	Nominated teacher	19.4
	Diploma-level Teacher	55.5

Data collection was carried out online, using the LimeSurvey Platform (LimeSurvey, v5). The supervisors of the study were employees of the Institute of Psychology and the Institute of Sociological Sciences of the John Paul II Catholic University of Lublin. The study was conducted in accordance with the guidelines of the Declaration of Helsinki and was approved by the Disciplinary Ethics Committee of the Institute of Sociological Sciences of the John Paul II Catholic University of Lublin (protocol code: 19/DKE/NS/2020).

### Statistical methods

**Fig 1:**
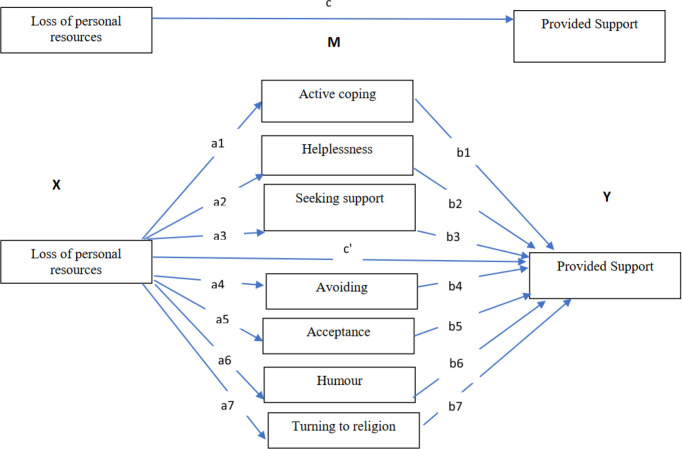
Conceptual diagram of the mediating roles of coping strategies for the relationship between loss of personal resources and providing support to students.

The data were analyzed with IBM SPSS Statistics for Windows, version 27 (IBM Corp.). Correlations between the variables were carried out, and then we examined whether the relationship between loss of personal resources and provided support to students is mediated by coping strategies (Fig 1). We analyzed the total effect of loss of personal resources and provided support (path c). The total effect is the sum of the direct effect (c’) of loss of personal resources on provided support while controlling for the mediator (coping strategies) and all indirect effects (paths a1-b1; a2-b2, a3-b3 etc.) of loss of personal resources on provided support by the mediating role of coping strategies. We performed an analysis using the SPSS add-on Process. The mediation analysis was conducted under the guidelines provided by Preacher and Hayes [56]. Following the bootstrapping (5000 bootstrapped samples) approach, indirect (mediated) effects whose confidence intervals (CI 95%) did not include zero were considered statistically significant. Our sample size (n = 2500) was sufficient to reach 0.8 power and to detect a significant mediating effect (Monte Carlo simulation).

## Results

Table 2 shows the correlation between the analyzed variables.

**Table 2:** Bivariate correlations between the variables included in the study (n = 2500).

Measure		1	2	3	4	5	6	7	8	9
MINI-COPE	[1] Active coping	–								
	[2] Helplessness	−0.239^***^	–							
	[3] Seeking support	0.323^***^	−0.054^**^	–						
	[4] Avoiding	−0.014	0.257^***^	0.182^***^						
	[5] Acceptance	0.371^***^	−0.079^**^	0.216^***^	0.085^***^	–				
	[6] Humour	0.152^***^	0.033	0.084^***^	0.180^***^	0.183^***^	–			
	[7] Turning to religion	0.100^***^	0.024	0.214^***^	0.126^***^	0.138^***^	0.060^**^	–		
Loss of personal resources (COR) [8]		−0.244^***^	0.244^***^	−0.082^***^	0.159^***^	−0.130^***^	−0.081^***^	0.070^***^	–	
Provided support (BSSS) [9]		0.329^***^	−0.236^***^	0.456^***^	0.019	0.215^***^	0.149^***^	0.184^***^	−0.178^***^	0.329^***^
M		2.43	1.03	2.11	1.75	2.13	1.27	1.49	12.16	3.21
SD		0.54	0.51	0.66	0.55	0.55	0.69	1.03	6.93	0.50

*** *< 0.001,*

** *< 0.01*

**Fig 2:**
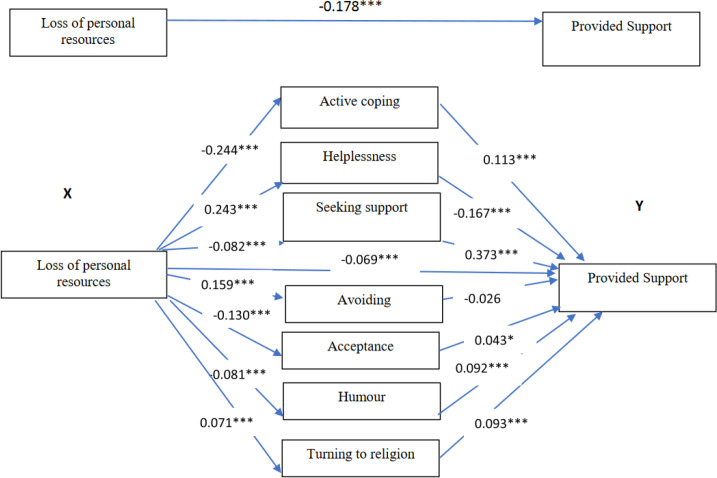
The mediating roles of coping strategies for the relationship between loss of personal resources and providing support to students in a group of teachers. ***<0.001, **<0.01, * <0.05; standardized coefficients.

In the whole group, the effect of loss of personal resources on providing support was partially mediated by active coping (a1 x b1), helplessness (a2 x b2), seeking support (a3 x b3), humour (a6 x b6) and turning to religion (a7 x b7). The total indirect effect was −0.109 (95% CI: −0.132; −0.087) (Tables 3, Fig 2).

**Table 3:** Standarized indirect effect of loss of personal resources on providing support to students through coping strategies in a group of teachers.

Mediator	BC 95% CI			
	Effect	SE	LL	UL
Active coping	−0.028	0.006	−0.039	−0.017
Helplessness	−0.041	0.006	−0.053	−0.030
Seeking support	−0.030	0.007	−0.045	−0.016
Avoiding	−0.004	0.003	−0.010	0.002
Acceptance	−0.006	0.003	−0.011	0.001
Humour	−0.007	0.002	−0.013	0.003
Turning to religion	0.007	0.002	0.003	0.011

BC, bias corrected; CI, confidence interval; SE, *standard error* estimate; LL, lower limit; UL, upper limit

**Fig 3:**
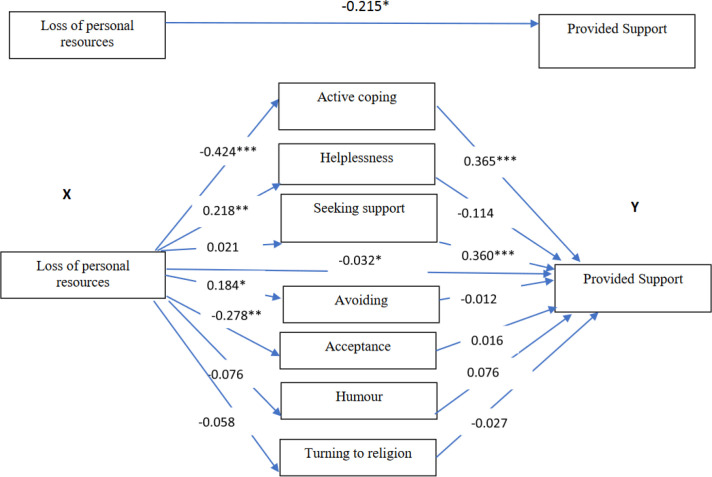
The mediating roles of coping strategies for the relationship between loss of personal resources and providing support to students in a group of non-believing teachers. ***<0.001, **<0.01, * <0.05; standardized coefficients.

In the group of non-believer teachers, the effect of loss of personal resources on providing support was partially mediated by active coping. In other words, active coping (a1 × b1) was significant mediators. The total indirect effect was −0.183 (95% CI: −0.304; −0.060) (Tables 4, Fig 3).

**Table 4:** Standarized indirect effect of loss of personal resources on providing support to students through coping strategies in a group of non-believing teachers.

Mediator	BC 95% CI			
	Effect	SE	LL	UL
Active coping	−0.155	0.048	−0.253	−0.066
Helplessness	−0.025	0.020	−0.069	0.009
Seeking support	−0.007	0.031	−0.053	0.071
Avoiding	−0.002	0.016	−0.035	0.030
Acceptance	−0.004	0.025	−0.059	0.043
Humour	−0.006		−0.035	0.013
Turning to religion	0.002		−0.104	0.019

BC, bias corrected; CI, confidence interval; SE, *standard error* estimate; LL, lower limit; UL, upper limit

**Fig 4:**
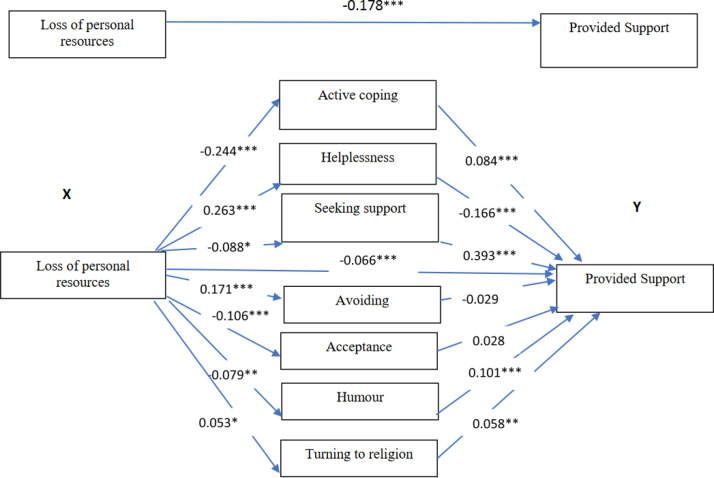
The mediating roles of coping strategies for the relationship between loss of personal resources and providing support to students in a group of believing teachers. ***<0.001, **<0.01, * <0.05; standardized coefficients.

In the group of believer teachers, the effect of loss of personal resources on providing support was partially mediated by active coping (a1 x b1), helplessness (a2 x b2), seeking support (a3 x b3), humour (a6 x b6) and turning to religion (a7 x b7). The total indirect effect was −0.113 (95% CI: −0.140; −0.084) (Tables 5, Fig 4).

**Table 5:** Standarized indirect effect of loss of personal resources on providing support to students through coping strategies in a group of believing teachers.

Mediator	BC 95% CI			
	Effect	SE	LL	UL
Active coping	−0.021	0.007	−0.034	−0.008
Helplessness	−0.044	0.007	−0.059	−0.030
Seeking support	−0.035	0.009	−0.053	−0.017
Avoiding	−0.005	0.004	−0.013	0.002
Acceptance	−0.003	0.003	−0.009	0.002
Humour	−0.008	0.003	−0.014	−0.003
Turning to religion	0.003	0.002	0.001	0.007

BC, bias corrected; CI, confidence interval; SE, *standard error* estimate; LL, lower limit; UL, upper limit

## Discussion

A discussion of the results will be presented in the context of the verification of the hypotheses. The first hypothesis was that the perception of higher levels of stress generated in teachers by personal losses is significantly associated with lower levels of the support provided to students. The presented correlations allowed the hypothesis to be positively verified. Namely, on the basis of the results obtained in the three models tested (model 1.: relationships occurring in teachers without taking into account their attitude to faith; model 2.: relationships occurring in teachers declaring themselves as non-believers; model 3.: regularities occurring in teachers declaring themselves as believers), it can be concluded that teachers’ perception of losses in personal resources co-occurs with a reduction in the support provided to pupils/ward. The relationships presented are in line with the results found in the literature [16,21–22,35]. The results obtained confirm two principles developed from COR theory – the principle of primacy of loss and the principle of desperation. According to the principle of primacy of loss, it can be concluded that the loss of personal resources is disproportionately more important for teachers’ functioning than their gain. At the same time, those who lack personal resources are more susceptible to subsequent resource losses and are less able to gain and/or rebuild them, which consequently generates higher stress. At the same time, the greater strength of perceived losses relative to resource gains leads to cycles of resource loss that increase in speed and magnitude over time, resulting in increasing levels of stress. A consequence of the mechanisms outlined is the regularity described in the fourth COR principle (principle of desperation). The essence of this principle is that depletion of resources makes it impossible or much more difficult to provide support to students [25,32–33].

The results of the study also confirmed the second hypothesis, which was that in the group of teachers, coping strategies act as mediators for the relationship between the stress generated by personal losses and the level of the support provided to students. In the model tested for the whole group of respondents (cf. model 1.), it was observed that five mediators had significant effects on relationship between the feeling of personal losses generating stress and the support provided to students (active coping, helplessness, support seeking, humour and turning to religion). The relationships present in the model allow several mediating mechanisms to be identified.

The first is that an increase in stress generated by resource losses co-occurs with a decrease in the coping strategies of humour, support-seeking and active coping, which in turn generate an increase in the support provided. The mediation mechanism outlined is consistent with two principles of COR theory – the primacy of loss principle and the principle of investment of resources. It can be concluded according to the primacy of loss principle that experiencing personal losses (i.a. in sense of control, organisational skills, communication skills, optimism, self-efficacy) triggers cycles of further losses in the form of less frequent use of coping strategies to strengthen student support – such as: a) sense of humour (objectifying occurring problems through jokes/jokes), b) support-seeking (ability to ask others for help when an individual cannot cope with occurring problems on his/her own), c) active coping (focusing efforts on solving problems and/or taking action to improve a disadvantaged situation). The mechanism shows that while increased stress from resource losses can reduce a teacher’s use of certain coping strategies, it paradoxically leads to a greater focus on supporting students. The teachers often have a strong sense of duty to their students. Even when they are unable to cope effectively with their own stress, this sense of duty can drive them to ensure that students still receive the support they need. This might involve putting aside their own well-being to focus more intensely on their students’ needs. This response is fuelled by their commitment to their students’ success and well-being, even when their positive coping strategies are compromised. The exemplification of the resource investment principle will only occur if resource losses are halted. In such a situation, there is a high probability of triggering a cycle of resource gains in the form of the use of active coping strategies (sense of humour, seeking social support, active coping), which in turn will trigger another cycle of gains in the form of a higher intensity of support provided. The findings indicate that while resource losses generally reduce supportive actions, there are scenarios where intense stress may paradoxically lead to an increase in certain coping behaviors among teachers. This dynamic highlights a possible extension of COR theory, suggesting that individuals in caregiving roles (such as parents, healthcare professionals, teachers, etc.) may prioritize others’ well-being even when their resources are low. Research conducted among teachers shows that during the pandemic, the most important aspects were maintaining contact with students and parents, ongoing teaching with classes, as well as providing emotional support to students [57–58].

The second mediating mechanism refers to the fact that higher levels of personal losses are indeed linked to feelings of helplessness, which at the same time induces a decrease in the support provided to students. This mechanism is an exemplification of the primacy of loss principle with its implications in terms of generating cycles of loss [53]. Namely, teachers’ experience of personal losses (i.a. in their sense of control, organisational skills, communication skills, optimism, self-efficacy) triggers the first cycle of losses involving the preference for the coping strategy in the form of helplessness (adopting a passive attitude towards the events that occur), and this type of behaviour, in turn, leads to a reinforcement of the initiated cycle of resource losses (weakening of students’ supportive actions). To guarantee that the education system functions properly and serves all students, it is crucial to link support services and equip educators with the help they need [59]

The third mediating mechanism, on the other hand, is related to the fact that experienced personal (stress-generating) losses intensify the use of a coping strategy in the form of a turning to religion, while at the same time the high frequency of preference for this coping strategy co-occurs with an increase in the support provided to students. The obtained regularities demonstrate that the mediating functions of the positive religious coping strategy exemplify two principles of COR theory with their implications – the resource investment principle and the paradox principle. The principle of resource investment indicates that teachers, despite the perceived loss of personal resources, activate positive religious coping with stress, which in turn reinforces resilience activities that involve providing support to students. Thus, it can be concluded that the use of a turn towards religion represents a kind of protection against personal losses, as its use/application, despite personal losses, results in the strengthening of supportive actions. At the same time, the preference for a turn towards religion as a way of coping with difficulties has a high value in the teachers’ personal evaluation, because, according to the paradox principle, the perception of resource gains (in this context, the preference for a positive religious coping strategy) increases in circumstances of resource losses. This implies the high intensity of personal losses (i.a. sense of control, organisational skills, communication skills, optimism, self-efficacy), promotes the teachers’ appreciation of the turning to religion as a way of coping with stress (i.a. through religious practices, prayer and/or meditation), which then triggers another cycle of resource gains – the strengthening of providing support to students.

However, the model did not find that avoidance and acceptance strategies had mediating functions for the relationship between experiencing stress-generating personal losses and the manifestation of the support provided to students. The conclusion presented is based on the fact that these strategies are significantly associated with experiencing personal losses (a high level of personal losses favours an increase in the intensity of the coping strategy in the form of avoidance and a decrease in the frequency of the use of the behaviour in the form of acceptance), but at the same time, no significant relationships were found between these strategies – avoidance and acceptance – and the severity of the manifestation of support provided to students. The literature indicates that teachers’ active approach to handling difficult and problematic situations, along with avoiding denial as a coping strategy, promotes work-related well-being—especially in the areas of social relationships and self-fulfillment [48].

The research conducted also provides the basis for positive verification of the hypothesis that assumed that in the group of non-believing teachers relative to the group of believing teachers, other coping strategies mediate the relationship between the intensity of stress generated by personal losses and the intensity of the support provided to students. In the case of teachers declaring themselves as non-believers (cf. model 2.), only one remedial strategy acts as a mediator for the relationship between the perceived personal losses initiating stress and the support provided to students. The mediating mechanism is that a high intensity of perceived personal losses generates a decrease in the intensity of active coping with stress, which in turn reinforces of student support. Interpreting the result in the context of COR theory, it can be concluded that it exemplifies the occurrence of two principles concerning the distribution of resources – the principle of primacy of loss and the principle of investment of resources [16,25]. Namely, stress resulting from personal losses (e.g., in sense of control, organisational skills, communication skills, optimism, self-efficacy) in non-believing teachers significantly correlates with a decrease in the use of active/task-based coping (triggering a primary cycle of resource losses), which in turn generates a decrease in the support provided to students (triggering a secondary cycle of resource losses). While resource losses are high, resource investment will only occur if these losses are effectively inhibited. Then, high levels of personal resources will increase the likelihood of a preference for active coping (triggering the primary cycle of resource gains), which in turn will translate into an enhancement of the support provided to students (triggering the secondary cycle of resource gains). Teachers using problem-focused strategies negatively correlate with stress and professional burnout [60]. COR theory emphasizes the difficulty of recovering resources after significant losses. The preventative strategies could be highly effective in reducing stress and preventing resource depletion among teachers. Regular training or workshops on resilience building, time management, and interpersonal communication may help teachers develop robust resources that act as buffers against future losses. By focusing on resource-building before significant losses occur, schools may reduce the prevalence of stress-related issues among teachers. In the group of non-believing teachers, the remaining tested strategies do not mediate the relationship between losses in personal resources and the support provided to students. An increase in the perceived loss of personal resources during stressful situations is associated with more frequent feelings of helplessness and a tendency to avoid the problem, while the use of acceptance-based coping strategies becomes less common. Moreover, actively seeking social support often leads to greater actual support received by students [61].

In the case of believing teachers (cf. model 3.), analogous mediating relationships were observed as in the whole group. The first mechanism relates to the mediating functions of three coping strategies: sense of humour, seeking social support and active coping. The mediating relevance of these strategies for the relationship between stress initiated by personal losses and the support provided to students, on the one hand, is due to the fact that the aforementioned madiators negatively correlate with perceived personal losses (high intensity of perceived personal losses co-occurs with their infrequent preference for them), and on the other hand, with positive correlations with the support provided to students. In the context of COR theory, it can be concluded that the mediating functions of the aforementioned remedial strategies are characterised by mechanisms embodied in the principles of the primacy of loss and investment in resources [3]. This means that, when personal loss-generated stress is high, believing teachers will significantly less often prefer active coping strategies in the form of humour, seeking social support and active coping (triggering the primary resource loss cycle). At the same time, the infrequent preference for the aforementioned coping strategies will co-occur with a reduction in the support provided to students (triggering the secondary cycle of resource loss). The mechanisms embodied in the principle of resource investment will occur in believing teachers when personal resources are functioning at a high level, meaning that the process of their loss is inhibited. Under such circumstances, high levels of personal resources will act as triggers for active coping strategies – sense of humour, seeking social support, active coping with stress (initiating a primary cycle of resource gains). In turn, the use of the aforementioned strategies will contribute to the support provided to students (initiation of a secondary cycle of resource gains) [25].

The second mediating mechanism in the group of believing teachers is characteristic of the mediating role played by the remedial strategy of manifesting helplessness in stressful situations. Namely, an increase in experienced personal losses is positively associated with more frequent feelings of helplessness (initiation of the first cycle of resource losses), which in turn translates into a decrease in the support provided to students (reinforcement of the initiated cycle of resource losses). The pattern of relationships between variables presented thus reflects the principle of primacy of loss in COR theory [25]. These findings underscore the need for interventions that help teachers in recognizing and breaking the helplessness cycle, potentially by fostering proactive coping skills that can buffer the effects of personal losses and restore their capacity to the support provided to students.

Yet another mediating function in the group of believing teachers is provided by the coping strategy of turning to religion. The mediating mechanisms are that believing teachers, despite perceived losses in personal resources, trigger positive religious coping with stress (triggering the primary cycle of resource gains), which in turn reinforces the resistance action of providing support to students (triggering the secondary cycle of resource gains). The mechanisms outlined are characteristic of the second principle of COR theory (resource investment). In doing so, experiencing resource gains when using a positive religious coping strategy contributes to the fact that it is viewed very positively by teachers because, according to the paradox principle (the third principle of COR theory), the perception of resource gains increases in stressful circumstances where resource losses are present [3]. For believing teachers, religious coping is an adaptive strategy that not only compensates for losses but also actively enriches their ability to cope with stress and support students. More specifically, this relationship was weaker in teachers with high positive religious coping. Moreover, research shows that positive coping with religious emotions moderates the relationship between stress and burnout. More specifically, this relationship is weaker in teachers with strong positive coping with religious problems [46,62].

In the group of teachers declaring themselves to be believers, avoidance and acceptance do not mediate the relationship between stress generated by resource losses and the support provided to students, as perceived resource losses correlate significantly with the aforementioned strategies (positive correlation with avoidance, and negative correlation with acceptance), while there are no significant correlations between the intensity of the aforementioned strategies (avoidance and acceptance) and actions in the form of support provision.

## Conclusion

The research conducted justifies the formulation of conclusions, which largely relate to the mechanisms developed on the basis of COR theory. It finds that higher levels of personal resource loss lead to a decrease in the use of active coping strategies, which can create cycles of further resource loss. However, during COVID-19, teacher support for students increased despite this trend, likely due to a strong sense of duty and responsibility teachers often feel toward their students. Frequent use of active coping strategies, particularly in low-loss situations, can foster resource gains. Perceived personal losses can generate feelings of helplessness, undermining support for students. Teachers experiencing resource losses may turning to religion as a coping strategy, which enhances student support and triggers a cycle of resource gains, aligning with the paradox principle where perceived gains become more valuable amidst losses. Among believing teachers, three mediating mechanisms were identified, while non-believers primarily exhibited only active coping. This suggests that religiosity allows for greater flexibility in coping strategies, which is essential for effective stress management [43,63]. The results presented justify the postulate that teachers should benefit from various forms of support to counteract personal losses that generate stress, including support of a religious nature [64–65].

Although the pandemic has formally ended, its psychological, social, and professional effects are still being felt. These include teacher burnout, changes in relationships with students, loss of resources (e.g., mental health and a sense of purpose at work), and the need to adapt to challenging working conditions. The comparison between believing and non-believing teachers highlights how different value systems influence the ability to support others. This is not an issue limited to the pandemic—it remains relevant in the context of any crisis or difficult professional situation.

### Research limitations

Further empirical analysis should be carried out on various groups to verify the mechanisms described on the basis of COR theory – among others, in terms of the emergence of cycles of resource losses and gains, taking into account the relationships contained in the basic principles of this concept – the principle of primacy of losses, the principle of investment in resources, the paradox principle and the principle of desperation [66–67]. In further research on the mediating functions of coping strategies (in teachers as well as in other professional groups), it is worth seeking answers to the following questions: To what extent do personality traits influence the preference for religious coping among believers? Do religious individuals exhibit a distinct coping style with stress that functions as a relatively stable, habitual personality trait, compared to other coping strategies that tend to be more flexible and variable?
